# Vasculoprotective Effects of Combined Endothelial Progenitor Cells and Mesenchymal Stem Cells in Diabetic Wound Care: Their Potential Role in Decreasing Wound-Oxidative Stress

**DOI:** 10.1155/2013/459196

**Published:** 2013-06-17

**Authors:** Supakanda Sukpat, Nipan Isarasena, Jutamas Wongphoom, Suthiluk Patumraj

**Affiliations:** ^1^Inter-Department of Physiology, Graduate School, Faculty of Medicine, Chulalongkorn University, Bangkok 10330, Thailand; ^2^Stem Cell and Cell Therapy Research Unit, Department of Pharmacology, Faculty of Medicine, Chulalongkorn University, Bangkok 10330, Thailand; ^3^King Chulalongkorn Memorial Hospital, Bangkok 10330, Thailand; ^4^Department of Physiology, Center of Excellence for Microcirculation, Faculty of Medicine, Chulalongkorn University, Bangkok 10330, Thailand

## Abstract

To investigate whether the combined endothelial progenitor cells (EPCs) and mesenchymal stem cells (MSCs) could enhance angiogenesis and wound healing in diabetic mice. Balb/c nude mice were divided into five groups, including a control group, diabetic group (DM), DM injected with 1 × 10^6^  cells MSCs, DM injected with 1 × 10^6^  cells EPCs, and DM injected with combined 0.5 × 10^6^  cells MSCs and 0.5 × 10^6^  cells EPCs. After seven weeks, the mice were anesthetized, and bilateral full-thickness excision skin wounds were made on the dorsorostral back. The percentage of wound closure in DM group decreased significantly than in control and all other treated groups on day 7 and day 14 (*P* < 0.005). On day 14, the percentage of capillary vascularity in combine-treated group was significantly higher than in DM (*P* < 0.005). In the present study, we have demonstrated that the combined EPCs and MSCs can increase vascular endothelial growth factor (VEGF) level and angiogenesis which resulted in reduced neutrophil infiltration, decreased malondialdehyde (MDA) levels, and enhanced wound healing in diabetic mice model.

## 1. Introduction

A diabetic wound is characterized by poor circulation and impaired angiogenesis, which contributes to nonhealing ulcers and may result in subsequent amputation. In addition, 84% of amputations among diabetic foot-ulcer patients have been reported [1–3].

Diabetes-induced endothelium and vascular dysfunction are closely associated with oxidative stress due to hyperglycemia caused by the excessive generation of reactive oxygen species (ROS). It has also been suggested that ROS can reduce the number and function of EPCs [4]. Consequently, EPC dysfunction may decrease the vascular regenerative potential of diabetic patients and thereby contribute to the pathogenesis of diabetic ulcers [4]. Moreover, high levels of superoxides in the diabetic wound may result in abnormalities in the number of local cytokines in the wound as well as reduce the production of growth factors, such as platelet-derived growth factor (PDGF), transforming growth factor-*β* (TGF-*β*), and vascular endothelial growth factor (VEGF), which contribute to the delay in wound healing [5].

Recently, several studies have suggested that MSCs can promote wound healing, angiogenesis, and the release of proangiogenic factors, including VEGF, basic fibroblast growth factor (bFGF) and stromal-derived factor-1 (SDF-1) [6–8]. Thus, this study aimed to examine the effect of combined EPCs and MSCs treatment on angiogenesis and wound healing in diabetic mice. In particular, we assessed the levels of ROS in the wound area where the EPCs and MSCs were injected. We hypothesized that the vasculoprotective effect of these combined EPCs and MSCs may be due to their ability to decrease ROS levels in the diabetic wound area.

## 2. Materials and Methods

### 2.1. Animal Preparation

Male Balb/c nude mice (7-8 weeks old, 20–25 g) were purchased from the National Laboratory Animal Center, Salaya Campus, Bangkok, Thailand. The experimental procedures were conducted according to the guideline of experimental animals by The National Research Council of Thailand and were approved by the ethics committee at the Faculty of Medicine, Chulalongkorn University. The mice were housed at room temperature (25 ± 3°C) with one mouse per cage and fed with standard chow and sterilized water. The mice were divided into five groups.

 Group 1: wound control group with implanted fibrin gel (control).
 Group 2: diabetic wound group with implanted fibrin gel (DM).
 Group 3: diabetic wound group with implanted fibrin gel and 1 × 10^6^ MSCs (DM + MSCs).
 Group 4: diabetic wound group with implanted fibrin gel and 1 × 10^6^ EPCs (DM + EPCs).
 Group 5: diabetic wound group with implanted fibrin gel and combined 0.5 × 10^6^ EPCs and 0.5 × 10^6^ MSCs (DM + EPCs + MSCs).

Animals in the diabetic groups were induced by an injection of streptozotocin (STZ, Sigma Chemical Co., USA) in citrate buffer, pH 4.5 (Sigma Chemical Co., USA) at a dose of 45 mg/kg i.p. daily for 5 days. Two weeks later, the glucose level was measured from tail-vein blood, and mice with glycemia (over 200 mg/dL) were selected for the study [9].

### 2.2. Preparation of Mouse MSCs

Mouse MSCs were cultured in Dulbecco's Modified Eagle's Medium (DMEM) containing 5% fetal bovine serum (FBS), 1x Pen-strep, and L-glutamine at 37°C, 5% CO_2_. The medium was changed every 2 or 3 days. The cells were maintained by the Stem Cell and Cell Therapy Laboratory.

### 2.3. Preparation of Human EPCs

Human EPCs were purchased from Lonza. The cells were plated on culture dishes coated with fibronectin (Gibco) and cultured in endothelial cell basal medium 2 (EBM-2, Lonza) at 37°C and 5% CO_2_. The medium was changed every 2 or 3 days. The cells were maintained by the Stem Cell and Cell Therapy Laboratory.

### 2.4. Preparation of Stem Cells and Fibrin Gel

The cells were washed with sterile phosphate-buffered saline (PBS) and then incubated with 2 mL 0.25% trypsin for 1 minute at 37°C. Next, 10 mL of culture medium was added to the plate to neutralize the trypsin activity. The cell suspension was transferred into a tube, and the number of cells was quantified using a microscope at 100x magnification. The cells were then centrifuged for 5 min at 1000 rpm at room temperature. The supernatant was removed, and the cell pellet was mixed with 30 *μ*L fibrinogen and 30 *μ*L thrombin per wound (the fibrin gel consisted of fibrinogen and thrombin and was obtained from Shanghai RAAS Blood Products Co. Ltd, China) [10]. 

### 2.5. Wound Model

Six to seven weeks after the streptozotocin injection, the mice were anesthetized by an intraperitoneal injection of sodium pentobarbital at a dose of 55 mg/kg and an alcohol swab was used on its dorsorostral back. Bilateral full-thickness excision skin wounds (0.6 × 0.6 cm^2^) were created on each side of the midline [8]. Each mouse received fibrin gel (control and DM), fibrin gel and 1 × 10^6^ MSCs (DM + MSCs), fibrin gel and 1 × 10^6^ EPCs (DM + EPCs), and fibrin gel with combined 0.5 × 10^6^ MSCs and 0.5 × 10^6^ EPCs (DM + EPCs + MSCs) on the wound bed. The wounds were then covered using Tegaderm (3M, USA). The EPCs and MSCs were maintained by the Stem Cell and Cell Therapy Research Unit at Chulalongkorn University. In this study, we would like to test the effect of human EPCs on diabetic wound (DM + EPCs group) and to compare with another reference that used only mouse MSCs [8], and also with the combined group. Therefore, we need to develop the diabetic model in nude mice, which demonstrate an immune deficiency.

### 2.6. Wound Analysis

Digital images of the wounds were taken at days 0, 7 and 14. The wound area was analyzed using digital image software analysis (Image Pro II 6.1). The percentage of wound closure (%WC) was calculated using the following equation: (area of the original wound − area of the actual wound)/area of the original wound × 100 [8].

### 2.7. Determination of Capillary Vascularity

At days 7 and 14 following wounding, the mice were anesthetized with an intraperitoneal (IP) injection of sodium pentobarbital at a dose of 55 mg/kg. The jugular vein was cannulated for injection of 0.2 mL of 5% FITC-labeled dextran (MW = 250000, Sigma Chemical Co., USA). The percentage of capillary vascularity (CV) was examined using intravital fluorescence video microscopy with a 10x objective lens [11]. From the video images, the percentage of capillary vascularity (%CV) was analyzed using Image Pro II 6.1 software. The region of interest (ROI) was 100 × 100 pixels. Each ROI was selected to cover only capillary networks with a diameter of less than 15 *μ*m [11, 12]. The average CV consisted of at least three ROIs from each animal. 

These results were confirmed by a blind assessment. The %CV was calculated using the following formula: (number of pixels within the capillaries/total number of pixels within the select area) × 100 [11, 12].

### 2.8. Measurement of Neutrophil Infiltration

The mice were sacrificed at 7 and 14 days, and the wound samples were harvested at a size of 0.6 × 0.6 cm^2^. The tissue specimens were fixed in 10% formaldehyde for 24 hours. The center of the wound samples was cut and embedded in paraffin. Four-micrometer thick sections were stained with hematoxylin-eosin (H&E). Neutrophil infiltration was measured using a microscope (Nikon E50i, Japan) at 400x magnification. The inflammatory wound area of the specimens was evaluated. The wound image was captured by a digital camera (Nikon DS-L2, Japan), and the amount of neutrophil infiltration was analyzed using Image Pro II 6.1 software [12]. These results were also confirmed by blind assessment.

### 2.9. Determination of Tissue VEGF, Malondialdehyde Levels, and Total Tissue Protein

The tissue sample was harvested from each mouse at 7 and 14 days following wounding and frozen at −80°C. A 50 mg tissue sample was homogenized in 50 mL RIPA lysis buffer (Cell Signaling Technology, Inc.) with protease inhibitor (Sigma Chemical Co., USA) and phosphatase inhibitor cocktails (Sigma Chemical Co., USA). The sample was sonicated and centrifuged at 10,000 rpm for 10 min. The supernatants of each tissue sample were used to analyze the levels of VEGF, malondialdehyde (MDA; lipid peroxides parameter), and the total tissue protein using ELISA (R&D Systems), TBARS assay kit (Cayman Chemical Co., USA) and microplate BCA protein assay kit (Thermo Scientific, USA), respectively. The VEGF and MDA levels were expressed as pg/mg protein and nM/mg protein, respectively.

### 2.10. Statistical Analysis

All of the data were presented as the mean ± SEM, and comparison between groups was conducted using one-way ANOVA, followed by the Least Significant Difference posttest (LSD). The correlation analysis was conducted using the two-tailed Pearson's correlation. Differences were considered statistically significant at a *P* value less than 0.05 (*P* < 0.05). 

## 3. Results and Discussion

The results of body weight and blood glucose are shown for each group (Table 1). On days 7 and 14, the blood sugar in the DM, DM + MSCs, DM + EPCs, and DM + EPCs + MSCs groups were significantly higher than their age-matched controls. The body weights of the all four groups of diabetes tend to decrease; however, they were not shown to be of statistical significance; that may be due to the small number of each group and high variability (Table 1).

### 3.1. Effects of EPCs and MSCs on Wound Closure

**Figure 1:**
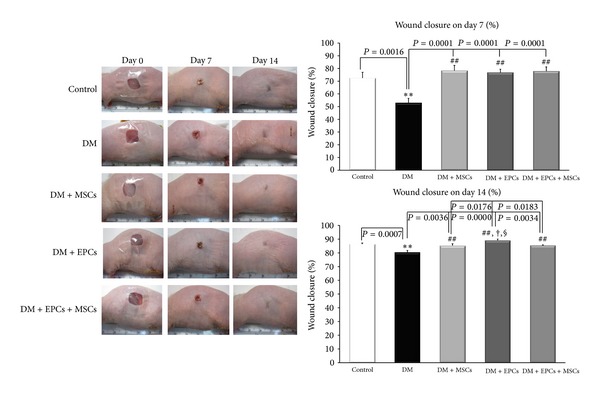
The percentages of wound closure in the control group with implanted fibrin gel (control), diabetic wound group with implanted fibrin gel (DM), diabetic wound group with implanted fibrin gel and MSCs (DM + MSCs), diabetic wound group with implanted fibrin gel and EPCs (DM + EPCs), and diabetic wound group with implanted fibrin gel and combined MSCs and EPCs (DM + MSCs + EPCs) on day 7 and day 14 are shown. The data are presented as the means ± SEM. **P < 0.005: significant difference compared to the control group. ^##^ *P* < 0.005: significant difference compared to the DM group. ^†^ *P* < 0.05: significant difference compared to the DM + MSCs + EPCs group. ^§^ *P* < 0.05: significant difference compared to the DM + MSCs group.

On day 7 and day 14 of postoperative wound, the percentage of wound closure (WC) in all of the treatment groups and control group was significantly increased compared to the DM group (*P* < 0.005; Figure 1). Interestingly, 14 days after wounding, the %WC of the DM + EPCs group was significantly increased compared to the DM + MSCs and DM + EPCs + MSCs groups (*P* < 0.05; Figure 1). 

### 3.2. Effects of EPCs and MSCs on Angiogenesis

**Figure 2:**
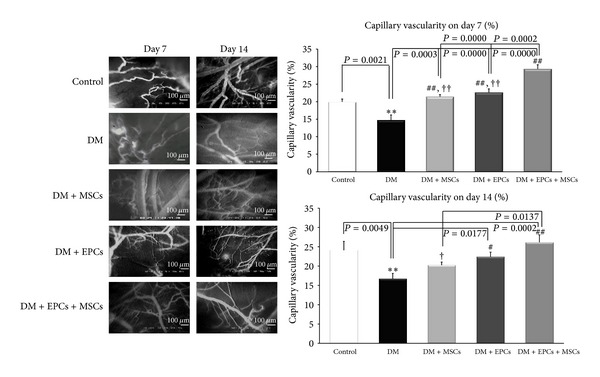
Percentages of capillary vascularity in the control group with implanted fibrin gel (control), diabetic wound group with implanted fibrin gel (DM), diabetic wound group with implanted fibrin gel and MSCs (DM + MSCs), diabetic wound group with implanted fibrin gel and EPCs (DM + EPCs), and diabetic wound group with implanted fibrin gel and combined MSCs and EPCs (DM + MSCs + EPCs) on day 7 and day 14 are shown. The data are presented as the means ± SEM. **P < 0.005: significant difference compared to the control group. ^#^ *P* < 0.05: significant difference compared to the DM group. ^##^ *P* < 0.005: significant difference compared to the DM group. ^†^ *P* < 0.05: significant difference compared to the DM + MSCs + EPCs group. ^††^ *P* < 0.001: significant difference compared to the DM + MSCs + EPCs group.

The results on day 7 showed that the %CV in all of the treatment groups and control group was significantly increased compared to the DM group (*P* < 0.005; Figure 2). Moreover, these results indicated that, on day 7 and day 14, the %CV of the DM + EPCs + MSCs group was significantly higher compared to the values of the DM and DM + MSCs groups (*P* < 0.001, *P* < 0.05; Figure 2). 

### 3.3. Effects of EPCs and MSCs on Tissue VEGF Levels

**Figure 3:**
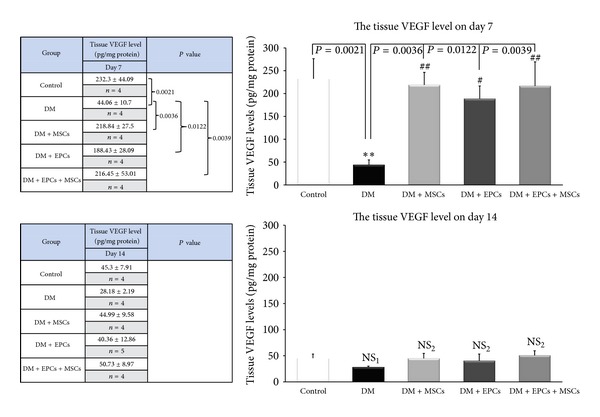
Effects of EPCs and MSCs on tissue VEGF levels. The tissue VEGF levels in the control group with implanted fibrin gel (control), diabetic wound group with implanted fibrin gel (DM), diabetic wound group with implanted fibrin gel and MSCs (DM + MSCs), diabetic wound group with implanted fibrin gel and EPCs (DM + EPCs), and diabetic wound group with implanted fibrin gel and combined MSCs and EPCs (DM + MSCs + EPCs) on day 7 and day 14 are shown. The data are presented as the means ± SEM. **P < 0.005: significant difference compared to the control group. ^#^ *P* < 0.05: significant difference compared to the DM group. ^##^ *P* < 0.005: significant difference compared to the DM group. NS_1_: no significant difference compared to the control group. NS_2_: no significant difference compared to the DM group.

The tissue VEGF levels of all of the treatment groups and control group were significantly increased compared to the DM group only on day 7 (Figure 3). There was no significant difference in the tissue VEGF levels among the groups on day 14.

### 3.4. Effects of Combined EPCs and MSCs on Neutrophil Infiltration

**Figure 4:**
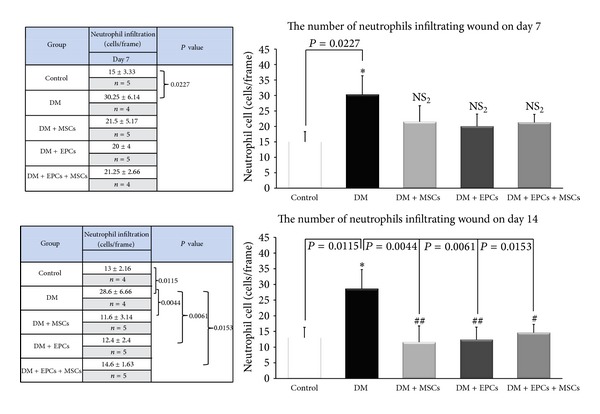
The number of infiltrating neutrophils in the control group with implanted fibrin gel (control), diabetic wound group with implanted fibrin gel (DM), diabetic wound group with implanted fibrin gel and MSCs (DM + MSCs), diabetic wound group with implanted fibrin gel and EPCs (DM + EPCs), and diabetic wound group with implanted fibrin gel and combined MSCs and EPCs (DM + MSCs + EPCs) on day 7 and day 14 is shown. The data are presented as the means ± SEM. *P < 0.05: significant difference compared to the control group. ^#^ *P* < 0.05: significant difference compared to the DM group. ^##^ *P* < 0.01: significant difference compared to the diabetic group. NS_2_: no significant difference compared to the DM group.

The number of neutrophils infiltrating into the wound area between the five groups is shown in Figure 4. On day 7, the number of infiltrating neutrophils in the DM group significantly increased compared to the control group (*P* = 0.02). Moreover, the DM + MSCs, DM + EPCs, and DM + EPCs + MSCs groups showed no significant difference when compared to the DM group (Figure 4).

On day 14, the number of infiltrating neutrophils in the DM group significantly increased compared to the control group (*P* = 0.011). The DM + MSCs, DM + EPCs, and DM + EPCs + MSCs groups showed a significantly reduced number of neutrophils compared to the DM group (*P* = 0.004, *P* = 0.006, *P* = 0.015, resp.); however, there was no significant difference among the DM + MSCs, DM + EPCs, and DM + EPCs + MSCs groups (Figure 4).

### 3.5. Effects of Combined EPCs and MSCs Treatment on Tissue MDA Levels

**Figure 5:**
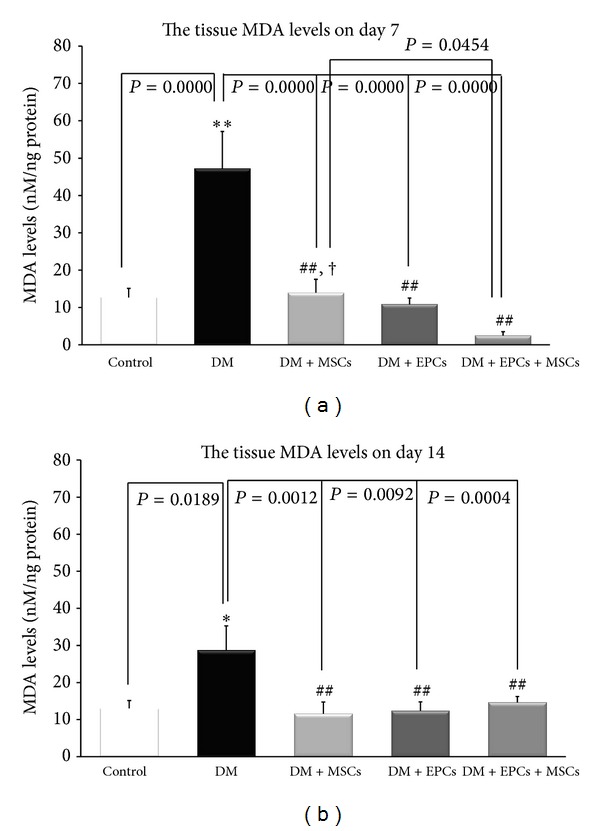
The tissue malondialdehyde levels (MDA) in the control group with implanted fibrin gel (control), diabetic wound group with implanted fibrin gel (DM), diabetic wound group with implanted fibrin gel and MSCs (DM + MSCs), diabetic wound group with implanted fibrin gel and EPCs (DM + EPCs) and diabetic wound group with implanted fibrin gel and combined MSCs and EPCs (DM + MSCs + EPCs) on day 7 and day 14 are shown. *P < 0.05: significant difference compared to the control group. **P < 0.0001: significant difference compared to the control group. ^##^ *P* < 0.01: significant difference compared to the DM group. ^†^ *P* < 0.05: significant difference compared to the DM + MSCs + EPCs group.

On day 7 and day 14 of postoperative wound, the tissue MDA levels in the DM group were significantly higher compared to all of the other groups (Figure 5). Interestingly, at 7 days after wounding, the tissue MDA levels in the DM + EPCs + MSCs group were significantly decreased compared to the DM and DM + MSCs groups (*P* < 0.01, *P* < 0.05; Figure 5). However, there was no significant difference between the DM + EPCs + MSCs and DM + EPCs groups.

In the present study, to induce type 1 diabetes mellitus in nude mice, multiple low doses of STZ (45 mg/kg body weight) were administered to the mice daily for 5 days. STZ chemically damages pancreatic *β* cells by DNA damage and results in hypoinsulinemia and hyperglycemia [13, 14]. As shown in the present study, the blood glucose levels in the DM, DM + MSCs, DM + EPCs, and DM + EPCs + MSCs groups were significantly higher than those in the controls for both of the studied periods (Table 1). These results also showed no effect of EPCs and MSCs on the changes in blood glucose levels.

Diabetes is characterized by the reduced production of growth factors, poor circulation, and impaired angiogenesis, which contribute to poor wound healing, which has been reported in diabetic patients. VEGF, an important growth factor that induces angiogenesis, was also reduced in the DM group [15]. The present results showed that on day 7, tissue VEGF levels were significantly increased in the DM + MSCs, DM + EPCs and DM + EPCs + MSCs groups compared to the DM group. However, no significant difference between all of the groups on day 14 was observed. According to the normal process of wound healing, increased VEGF is normally increased maximally on day 7 and then declines at day 14 after the emergence of neocapillaries [16].

Taken together, these results also suggested that combined EPCs and MSCs have the potential to increase VEGF similar to the DM + EPCs and DM + MSCs groups. There was also no significant difference among the treatment groups on day 7.

It is thought that MSCs can increase chemoattractive and mitogenic factors, including VEGF, similar to EPCs [6–8]. Moreover, it has been reported that MSCs may also increase the number of cells positive for CD34, C-kit, or Flk-1, which are endothelial lineage cell markers and increase the recruitment of endothelial cells and endothelial progenitor cells into the wound [17–21]. As shown in our study (Figure 2), the combined DM + EPCs + MSCs group significantly demonstrated the highest increase in capillary vascularity on both day 7 and day 14. This result may be the first in vivo evidence indicating the synergistic effect of these two cell types on enhancing angiogenesis in the diabetic wound.

The present study also demonstrated that, on day 14, the number of infiltrating neutrophils in the DM + MSCs, DM + EPCs, and DM + EPCs + MSCs groups was decreased compared to the DM group (Figure 4).

Taken together, the results of the present study indicated that the combined treatment of EPCs and MSCs could reduce neutrophil infiltration, improve angiogenesis, and increase VEGF levels, which could promote a better outcome of wound closure in a diabetic mice model.

These results indicated that tissue MDA levels in the DM group were significantly increased compared to the control group. Combined EPC and MSC treatment significantly demonstrated the best potential in reducing the extent of wound-induced oxidative stress on day 7. The increase in ROS is a common characteristic at the wound site. In a previous study performed using an excision wound mouse model, the major product of lipid peroxidation, 4-hydroxy-2-nonenal (4-HNE), was reported [22]. Interestingly, coimmunostaining revealed that 4-HNE mainly colocalized with neutrophils; thus, it was suggested that the respiratory burst of these inflammatory cells results in the production of superoxide, which in turn causes lipid peroxidation.

Interestingly, low levels of ROS are required for the defense against invading pathogens [23], as well as for mediating intracellular signaling [24]. However, excessive amounts of ROS are deleterious due to their high reactivity. 

These results suggested that combined EPC and MSC treatment could improve the wound healing process due to their consequential effects on angiogenesis and anti-inflammation, resulting in a decrease in antioxidative stress. We proposed that combined EPC and MSC treatment could inhibit diabetes-induced oxidative stress in the wound area via two potential mechanisms. The first mechanism involves the promotion of sufficient blood perfusion to enhance angiogenesis in the wound area by combined treatment with EPCs and MSCs. Thus, hypoxia cannot amplify the early inflammatory response, thereby inhibiting prolonged injury. The second mechanism is that combined EPC and MSC treatment inhibits oxidative stress produced from the inflammatory cells, thereby restoring the balance between ROS and the antioxidant capacity within the wound area. 

**Figure 6:**
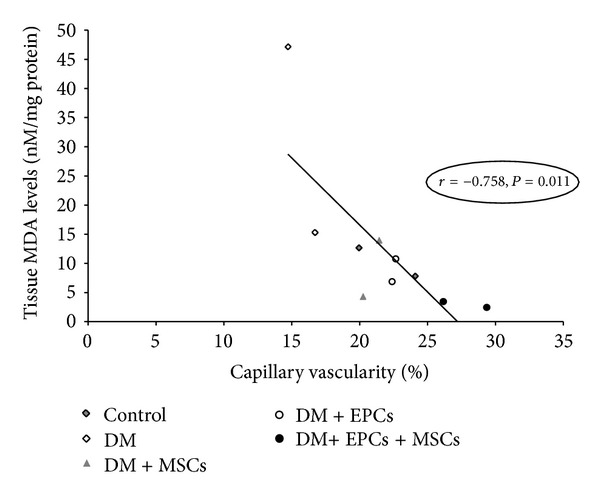
Relationship between capillary vascularity (%CV) and tissuemalondialdehyde levels (MDA). Data were obtained from the mean of each group on day 7 and day 14.

**Figure 7:**
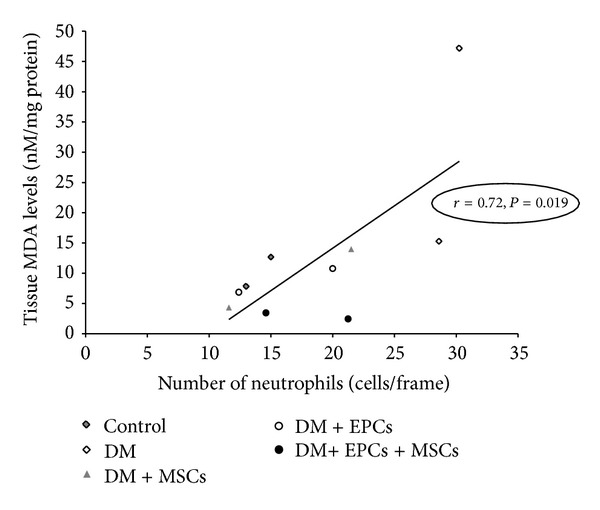
Relationship between number of neutrophils (Cells/frame) and tissuemalondialdehyde levels (MDA). Data were obtained from the mean of each group on day 7 and day 14.

Correlation analyses between tissue MDA levels and %CV and between MDA levels and the extent of neutrophil infiltration strongly support these two potential mechanisms (Figures 6 and 7). This correlation indicated that, in the treatment groups, the %CV increased with the reduced number of infiltrating neutrophils and significantly corresponded to the changes in wound ROS content, which is reflected at the MDA level.

Taken together, we showed that combined EPC and MSC administration significantly improved vascular function in diabetic wound mice. These data may have important implications for the potential use of MSCs as a cellular therapy and reduce the use of EPCs in diabetic wound conditions characterized by compromised vascularity and wound closure. Our study demonstrated that combined EPC and MSC treatment might provide benefits for diabetic patients and lower the risk of organ loss.

## Figures and Tables

**Table 1:** The means ± SEM of the body weight and blood glucose on day 7 and 14 after wounding in the wound control group with implanted fibrin gel (control), diabetic wound group with implanted fibrin gel (DM), diabetic wound group with implanted fibrin gel and 1 × 10^6^ MSCs (DM + MSCs), diabetic wound group with implanted fibrin gel and 1 × 10^6^ EPCs (DM + EPCs), and diabetic wound group with implanted fibrin gel and combined 0.5 × 10^6^ EPCs and 0.5 × 10^6^ MSCs (DM + EPCs + MSCs).

Group	Body weight (g)		Blood glucose level (mg/dL)	
	Day 7	Day 14	Day 7	Day 14
Control	25.91 ± 1.22	26.06 ± 1.45	115.17 ± 14.28	120.40 ± 11.52
DM	23.86 ± 2.07	24.77 ± 0.72	347.83 ± 59.41**	362.83 ± 57.56**
DM + MSCs	21.81 ± 1.40	22.61 ± 2.32	361.33 ± 18.63**	425.00 ± 104.74**
DM + EPCs	24.76 ± 1.41	22.86 ± 1.30	344.17 ± 61.21**	362.40 ± 35.56*
DM + EPCs + MSCs	24.57 ± 1.07	23.60 ± 0.71	312.33 ± 15.05**	376.50 ± 82.19*

*P < 0.05: significant difference compared to control group.

**P < 0.01: significant difference compared to control group.

## Abbreviations

bFGF: Basic fibroblast growth factor
DM: Diabetes mellitus
DMEM: Dulbecco's Modified Eagle's Medium
EBM-2: Endothelial cell basal medium 2
EPCs: Endothelial progenitor cells
FBS: Fetal bovine serum
MDA: Malondialdehyde levels
MSCs: Mesenchymal stem cells
PBS: Phosphate-buffered saline
PDGF: Platelet-derived growth factor
ROI: Region of interest
ROS: Reactive oxygen species
SDF-1: Stromal-derived factor-1
STZ: Streptozotocin
TGF-*β*: Transforming growth factor-*β*
VEGF: Vascular endothelial growth factor
%WC: Percentage of wound closure
%CV: Percentage of capillary vascularity
4-HNE: 4-Hydroxy-2-nonenal.
